# Hierarchical Porous, N-Containing Carbon Supports for High Loading Sulfur Cathodes

**DOI:** 10.3390/nano11020408

**Published:** 2021-02-05

**Authors:** Jae-Woo Park, Hyun Jin Hwang, Hui-Ju Kang, Gazi A. K. M. Rafiqul Bari, Tae-Gyu Lee, Byeong-Hyeon An, Sung Yong Cho, Young-Si Jun

**Affiliations:** 1 Department of Advanced Chemicals & Engineering, Chonnam National University, 77 Yongbong-ro, Buk-gu, Gwangju 61186, Korea; jaewoopark0218@gmail.com (J.-W.P.); grafiqulbari@gmail.com (G.A.K.M.R.B.); dlxorb007@gmail.com (T.-G.L.); 2 School of Chemical Engineering, Chonnam National University, 77 Yongbong-ro, Buk-gu, Gwangju 61186, Korea; wgguswls@gmail.com (H.J.H.); byeonghyeon2021@gmail.com (B.-H.A.); 3 Department of Environment and Energy Engineering, Chonnam National University, 77 Yongbong-ro, Buk-gu, Gwangju 61186, Korea

**Keywords:** lithium–sulfur batteries, high loading sulfur cathodes, hierarchical porous structures, pyridinic N functional groups, molecular cooperative assemblies, reactive templates

## Abstract

The lithium-polysulfide (LiPS) dissolution from the cathode to the organic electrolyte is the main challenge for high-energy-density lithium-sulfur batteries (LSBs). Herein, we present a multi-functional porous carbon, melamine cyanurate (MCA)-glucose-derived carbon (MGC), with superior porosity, electrical conductivity, and polysulfide affinity as an efficient sulfur support to mitigate the shuttle effect. MGC is prepared via a reactive templating approach, wherein the organic MCA crystals are utilized as the pore-/micro-structure-directing agent and nitrogen source. The homogeneous coating of spherical MCA crystal particles with glucose followed by carbonization at 600 °C leads to the formation of hierarchical porous hollow carbon spheres with abundant pyridinic N-functional groups without losing their microstructural ordering. Moreover, MGC enables facile penetration and intensive anchoring of LiPS, especially under high loading sulfur conditions. Consequently, the MGC cathode exhibited a high areal capacity of 5.79 mAh cm^−2^ at 1 mA cm^−2^ and high loading sulfur of 6.0 mg cm^−2^ with a minor capacity decay rate of 0.18% per cycle for 100 cycles.

## 1. Introduction

Grid-scale energy systems and electric vehicles require high-energy-density batteries. The current-state-of-the-art energy storage technology, i.e., lithium-ion batteries (LIBs), has already reached its theoretical limit [1,2]. To become more competitive with LIBs, the next-generation battery technologies must consider energy density, power density, cycle performance, and cost. Among the various energy storage systems, rechargeable lithium–sulfur batteries (LSBs) are widely considered as one of the most promising candidates [3,4]. Notably, LSBs have a high theoretical energy density (~2500 Wh kg^−1^ or ~2800 Wh L^−1^) and are also cost-effective because they use inexpensive, abundant, and eco-friendly elemental sulfur (S_8_) as their cathode active material [5]. In addition, the same manufacturing process now existing for LIBs can be employed for the LSBs [6]. Given the operating voltage of ~2.1 V vs. Li/Li^+^, the practical LSBs to replace the commercial LIBs of 2–4 mAh cm^−2^ require an areal capacity of 3–7 mAh cm^−2^, corresponding to an areal sulfur loading of 3–7 mg cm^−2^ at a sulfur utilization efficiency of 60%. The typical efficiency of such high-sulfur-loading LSB cells is, however, below 50% even at initial cycles, which quickly degrades to a lower value during cycling. This is attributed to (1) volume expansion (~180%) of sulfur during discharging, which induces pulverization of cathode composite; (2) lithium-polysulfide (LiPS) dissolution into the electrolyte and the corresponding shuttle effect, which diminishes cycle stability and Coulombic efficiency (CE); and (3) the final products (S_8_ and Li_2_S) during charging and discharging procedure, which, respectively, are fundamentally electronic/ionic insulating leading to large polarization and cell degradation [7,8,9].

A representative effort, which has been proven to be highly efficient at improving the electrochemical performance of the high sulfur loading LSBs, includes the development of multi-functional sulfur host materials featuring (1) porous structure exerting capillary force and accommodating volume expansion during the lithiation of sulfur, (2) high electrical/ionic conductivity for efficient redox reaction of S_8_ and Li_2_S, and (3) a catalytic active site for specific chemical interaction with LiPS [10,11,12]. Among the various sulfur host materials such as transition metal oxide/nitride/sulfide, metal-organic frameworks (MOFs), conductive polymer, carbon-based materials, etc., carbon-based materials are of particular interest [13]. They can be synthesized from an inexpensive precursor and feature tailorable structural/electronic properties. In particular, hierarchical porous carbon (HPC) is very attractive in view of the systematic multi-pore structures of micro-/meso-/macro-pores, which enable efficient polysulfide retention, mass diffusion/transport, and accommodation of volume expansion, respectively [14]. The main challenges in the HPC materials for LSBs include the counterbalance among pore-, micro-, and chemical structure as well as the complicated synthesis procedures using harmful chemicals. For example, increasing porosity and heteroatom-doped in carbon materials deteriorate the microstructure and thus the electronic conductivity, leading to poor chemical performance, especially under high sulfur loading conditions. In addition, high porosity often decreases the tap density and thus volumetric energy density of the electrode, rendering the porous carbon-based LSBs impractical in comparison to the state-of-the-art LIBs.

In general, the tunable physical/chemical properties of the carbon matrix can be achieved by simply employing different temperatures, gas environments, and precursors. They introduce different carbonization modes and thereby modify the carbonization intermediates/products in terms of the degree of aromatic condensation, quality of graphitic crystallites, and chemical composition, wherein the electronic properties of carbon materials are dictated [15]. Such tunability would also facilitate the systematic studies toward revealing the microstructure, chemical composition, and electrochemical function correlated to LSBs. Herein, we demonstrated in this work that MCA-glucose-derived carbon (MGC) with both high porosity and electronic conductivity is beneficial for polysulfide retention, mass-transfer of electrolyte and active species, and accommodation of volume change (180%) from elemental sulfur to Li_2_S during the discharge process. The MGC contains mass-producible spherical carbon particles with three-dimensional interconnected arrays and a hierarchical porous structure of micro-, meso-, and macro-pores. More importantly, it achieves a high content (~10 wt.%) of nitrogen by using a melamine-cyanuric acid network of MCA as a reactive template, strengthening affinity to polysulfide dissolved in organic electrolyte solvent, with little affecting the microstructure and electronic properties of MGC. Thus, a cathode for the high sulfur-loading was successfully fabricated using the MGC for high areal capacity LSBs. The micro-structure, pore-structure, and chemical composition of the carbon supports showed an areal capacity of 5.79 mAh cm^−2^ at areal 6.0 mg_Sulfur_ cm^−2^ and achieved a minor capacity decay rate of 0.18% per cycle by simple carbonization of the mixture of glucose and molecular cooperative assembly of the organic crystals. The morphologies, porosity, lateral size of graphite-like crystallite, and nitrogen content of the MGC were comprehensively evaluated. Thereafter, the structure-electrochemical performance correlation was investigated by galvanostatic cycling with potential limitation (GCPL), cyclic voltammetry (CV), and electrochemical impedance spectroscopy (EIS).

## 2. Materials and Methods

### 2.1. Materials

Melamine (99%), cyanuric acid (98%), 1,3-dioxolane (DOL, 99.8%), 1,2-dimethoxyethane (DME, 99.5%), lithium bis(trifluoromethylsulfonyl)imide (LiTFSI, 99.5%), and sulfur (99.5%) were purchased from Sigma-Aldrich (Darmstadt, Germany). Lithium sulfide (Li_2_S, 99.9%), lithium nitrate (LiNO_3_, 99.99%), and lithium metal (99.9%) were purchased from Alfa Aesar (Haverhill, MA, USA). N-methyl pyrrolidone (NMP, 99.0%, DAEJUNG Co., Siheung, Korea), polyvinylidene fluoride (PVdF, MTI Co., Richmond, CA, USA), Super P (Timcal Co., Bodio, Switzerland), dimethyl sulfoxide (DMSO, 99.5%, DAEJUNG Co., Siheung, Korea), glucose (98%, JUNSEI Co., Tokyo, Japan), and ethanol (94.5%, DAEJUNG Co., Siheung, Korea) were also used. All the reagents were purchased commercially and used without purification.

### 2.2. Synthesis of MGC and GLC

MCA was synthesized using a previously reported method [16]. Glucose (1 g) was totally dissolved in ethanol (10 mL) and triple-distilled water (10 mL) by sonication for 1 h. Thereafter, MCA (1 g) was added to the glucose solution and stirred overnight. The mixed powder was obtained by the evaporation of the mixed solution at room temperature in a fume hood (>1.0 m s^−1^). Next, MCA-glucose carbon (MGC) was carbonized at 600 °C with 2.3 °C min^−1^ and retained for 4 h in an inert N_2_ atmosphere. The glucose carbon (GLC) was prepared at the same carbonization process, without MCA.

### 2.3. Characterization

An X-ray photoelectron spectroscopy (XPS, K-ALPHA) from Thermo Scientific, Waltham, Massachusetts, United States, was employed to analyze the material’s chemical state and elemental composition using Al monochromatic X-ray source. The extent of nitrogen sorption of the material was quantified by sorption isotherm using an instrument of Micromeritics TriStar II 3020 analyzer from Norcross, Georgia, United States at −196 °C. The materials were pre-treated by deaeration at 150 °C for 12 h before the trigger of the analysis. A 3D high-resolution X-Ray Diffractometer (XRD, EMPYREAN, Malvern Panalytical, Malvern, United Kingdom) was used to analyze the crystal structure of the materials by Cu Kα radiation (λ = 1.54056 Å). A laser Raman spectrophotometer (NRS-5100, JASCO Inc., Easton, MD, USA) was utilized to assume the material’s structural footprint precisely using the laser excitation line at 532 nm. The material’s morphological characterization was conducted by an FE-SEM (Field emmision scanning electron microscope, HITACHI, SU-70, Tokyo, Japan) and HR-TEM (high-resolution transmission electron microscope, FEI Company, TECNAI F20 UT, Hillsboro, OR, USA).

### 2.4. Electrochemical Measurements

Initially, carbon, Super P, and polyvinylidene fluoride (PVdF) were mixed (80:10:10, *w*/*w*/*w*). Thereafter, the mixture was dispersed on NMP followed by sonication for 1 h and stirring overnight. Finally, the slurry was evenly drop-casted on a carbon cloth (CC) and dried at 120 °C for 24 h.

For blank electrolyte, 1 M lithium bis(trifluoromethanesulfonyl) imide (LiTFSI) was mixed in 1,3-dioxolane (DOL) and 1,2-dimethoxyethane (DME) (1:1; *v*/*v*) with 1 wt.% lithium nitrate (LiNO_3_). to make a blank electrolyte. Elemental sulfur and Li_2_S were mixed with blank electrolyte to make the catholyte (1 M Li_2_S_6_). The cells were assembled in an argon-filled glove box from Koreakiyon Company, Korea. A CR-2032 coin-type cell with a lithium foil and separator (MTI Co., Richmond, CA, USA) was used to assemble the cell.

GCPL tests were conducted using WMPG 1000S from WonATech, Korea (voltage range: 1.8–2.6 V vs. Li/Li^+^; current density: 1 mA cm^−2^). According to the experimental condition, the mass loading of sulfur was maintained at either 2.0 mg cm^−2^ or 6.0 mg cm^−2^. A VSP Biologic potentiostat instrument from Biologic company, France, was used for CV (voltage range: 1.8–2.6 V vs. Li/Li^+^) and EIS (frequency range: 250 kHz–50 mHz) measurements.

Capacity decay rate per cycle was calculated as follows. *Q_m_*, *Q_f_*, *C_m_*, and *C_f_* correspond to maximum capacity, final capacity, cycle number at maximum capacity, and final cycle number, respectively.

Capacity decay rate per cycle= Qm−QfQm×100Cf−Cm

## 3. Results

**Scheme 1:**
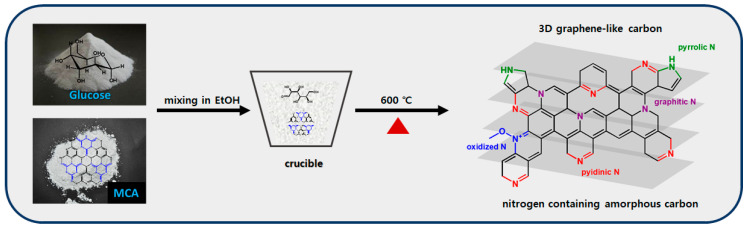
Nitrogen-containing hierarchical porous carbon preparation strategy.

**Figure 1:**
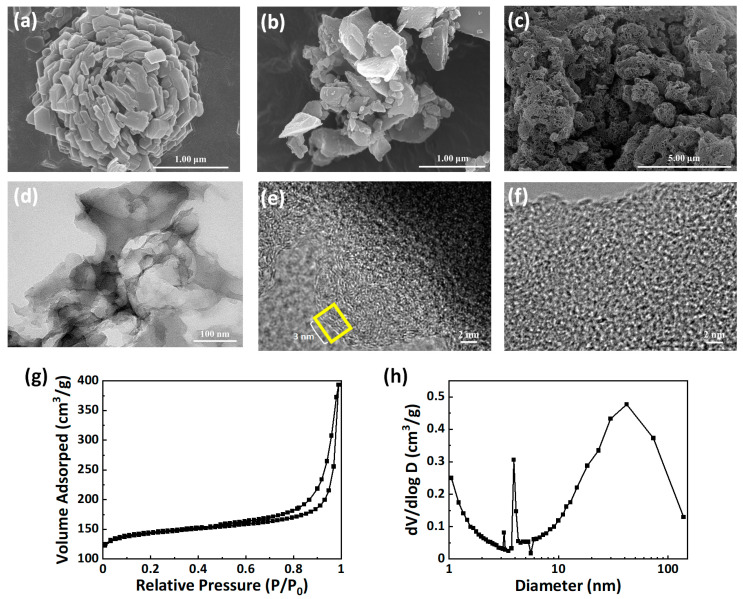
Scanning electron microscope (SEM) images of (**a**) MCA, (**b**) GLC, (**c**) MGC, transmission electron microscope (TEM) images of (**d**,**e**) MGC, (**f**) GLC, N_2_ adsorption–desorption results of MGC (**g**) isotherm profile and (**h**) Barrett-Joyner-Halenda (BJH) desorption pore size distribution.

A molecular cooperative assembly-mediated synthesis method was used to generate porous N-containing carbon supports without sacrificing the structural order (or integrity). A 1:1 hydrogen-bonded complex between melamine and cyanuric acid (MCA), the so-called reactive template, was used as a structure-directing agent as well as a nitrogen source for MGC (Figure 1a) [15]. According to the previously reported procedure, MCA was combined with an additional carbon source, i.e., glucose, via simple solution mixing in EtOH followed by carbonization at 600 °C to generate MGC in a gram scale (Scheme 1).

Glucose forms irregular rectangular chunks (GLC) ranging from 100 nm to 1 µm without porous structures at 600 °C (Figure 1b). Such MGC particles are completely transformed into interconnected uniform hollow spheres (MGC) of 1–2 µm, thereby demonstrating the successful templating of MCA by glucose (Figure 1c). Furthermore, MGC spheres essentially consist of thin graphene-like particles of 200–300 nm, matching well with the size of MCA hexagonal crystals, where macropores of 100 nm seen in SEM and TEM images result from the structural shrinkage during high-temperature polyaromatic condensation (Figure 1d). The HR-TEM image reveals the well-developed, ordered graphitic crystallites of 2–3 nm with abundant interstitial voids of 1.2 nm on the edge site, which is in marked contrast to the GLC (Figure 1e,f). XRD analyses combined with the Scherrer equation estimate that the graphitic crystallites have a lateral size of 2.72 nm and a stacking height of 0.92 nm for MGC, indicating the superior structural ordering of MGC compared to GLC. N_2_ sorption analysis shows that the interstitial voids and macropores contribute a high Brunauer-Emmett-Teller surface area and pore volume of 400 m^2^ g^−1^ and 0.51 cm^3^ g^−1^, respectively (Figure 1g,h).

Glucose is wrought as a microporous and highly aromatic condensed carbonaceous material with reactive templating MCA [15]. Most significantly, a temperature of 600 °C is maintained for the carbonization of the precursors to remove carbon nitride formed at 550 °C from the MCA on the carbon materials. If not, carbon nitride restrains electric conductivity because of its structural nitrogen content. It has been previously revealed that glucose is successfully carbonized on MCA’s surface, maintaining its parental morphology, rosette-like hollow sphere, and introducing porous structure, which might be beneficial for the retention of lithium-polysulfide (LiPS). Moreover, during the condensation of MCA, nitrogen, which is considered to have an affinity with the sulfur content in LSBs, will be doped easily in the resulting carbonaceous material and prevent the polysulfide shuttle effect.

**Figure 2:**
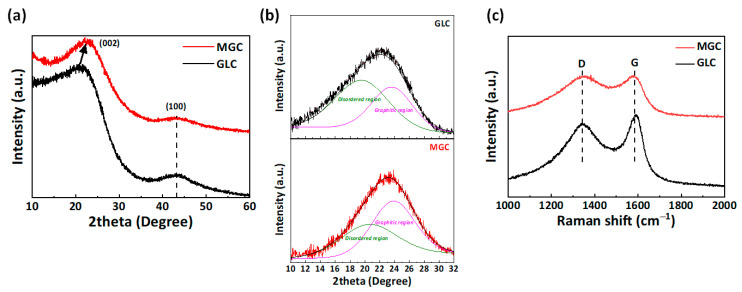
(**a**) XRD patterns of GLC and MGC, (**b**) deconvolution of the (002) peaks of GLC and MGC XRD patterns, and (**c**) Raman spectra of GLC and MGC.

Powder XRD analysis was carried out to investigate the microstructural characteristics of MGC (Figure 2a). One obvious change is that the peak corresponding to the (002) reflection, which represents the stacking distance between two-dimensional (2D) graphitic layers, is shifted from 0.42 nm (GLC) to 0.40 nm (MGC). In addition, according to Table 1, as calculated from the XRD patterns of the samples using Scherrer equation and Bragg’s law, MGC has a smaller size of the *c*-axis crystallite (L_c_ = 0.92 nm), larger lateral size (L_a_ = 2.72), and increased R factor (1.91) than those of GLC (L_c_ = 1.22 nm, L_a_ = 2.45, and R factor = 1.85). These factors indicate that the MGC is a few-layered, graphitized carbon with a higher degree of aromatic condensation in a 2D fashion. The homogeneous coating of glucose on 2D MCA sheets induces the microstructure-controlled carbon. The higher degree of aromatic condensation in MGC is further evidenced by the deconvolution of the (002) reflection peak into two peaks: one is a disordered region and the other is a graphitic region, similar to the previous work (Figure 2b) [15]. Notably, MGC is composed of a much more areal contribution of the graphitic region (50.34%) than that of GLC (40.94%), indicating a higher degree of aromatic condensation. To further evaluate the degree of graphitic in carbon materials, a Raman spectroscopy assay was conducted to calculate the I_G_/I_D_ ratio, wherein the lower the density of the defects, the higher the I_G_/I_D_ ratio (Figure 2c). The corresponding G-vibration peak centered on 1580 cm^−1^ corresponds to the sp^2^-hybridized carbon, and D-vibration peak on 1350 cm^−1^ corresponds to the sp^3^-carbon, indicating the defect region in a locality to sp^2^-carbon. Previous work revealed that the nitrogen-containing carbon prepared from MCA-glucose mixtures as a precursor showed a higher I_G_/I_D_ ratio than that of the bulk counterpart [15]. This is due to the presence of reactive template MCA, where the polyaromatic condensation of carbonaceous is more favorable on the 2D-organized sheet-like structure of MCA. However, this result is contrary to the previous one because of the vulnerability of the bulk counterpart, as the in-situ decomposition of the carbon-based structure and further graphitization occurred during Raman spectroscopy even by the visible light (532 nm), increasing the intensity of the G-vibration peak [17].

**Figure 3:**
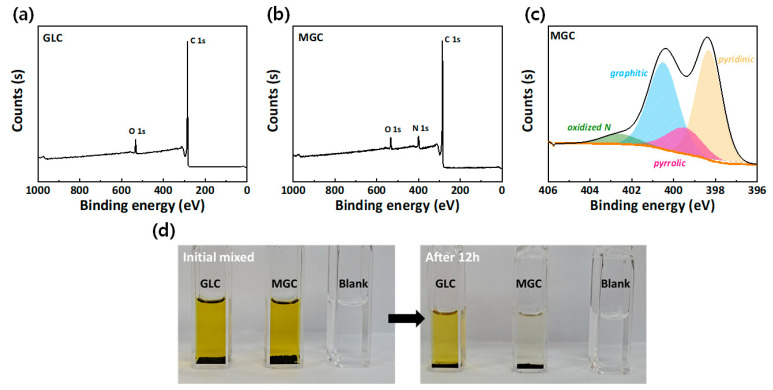
X-ray photoelectron spectroscopy (XPS) survey scan of (**a**) GLC and (**b**) MGC, (**c**) high-resolution N 1s spectrum of MGC, and (**d**) adsorption test of GLC and MGC in 2.5 mM Li_2_S_6_ solution.

Unlike GLC, the reactive template MCA endows MGC a high N content of 11.69% (Figure 3a,b Table 2). Identifying those species is important because of their characteristics, which affect the chemical and electronic properties of carbon materials, where the pyridinic N and graphitic N enable to increase affinity to the polysulfide and the electrical conductivity of the carbon-based material, respectively [18]. The high-resolution N 1s spectrum of MGC could be deconvoluted into four peaks at 398.3, 399.5, 400.5, and 402.6 eV corresponding to pyridinic, pyrrolic, graphitic, and oxidized N atoms, respectively (Figure 3c) [19,20]. MGC contains substantial amounts of graphitic N (38.82%), which is responsible for the increased I_G_/I_D_ ratio in Raman spectra [15]. In addition, the nitrogen in MGC is mainly composed of pyridinic nitrogen (41.98%), expecting the capability to quench the polysulfide shuttling effect. Indeed, it was shown that MGC could adsorb most of the 2.5 mM Li_2_S_6_ in the electrolyte solution (DOL:DME = 1:1 *v*/*v*) within 12 h, whereas GLC adsorbs a small amount of Li_2_S_6_ within the same time (Figure 3d). MGC as sulfur host material having increased the surface area and the amount of nitrogen contents obviously shows the improved anchorage of intermediated lithium polysulfide.

**Figure 4:**
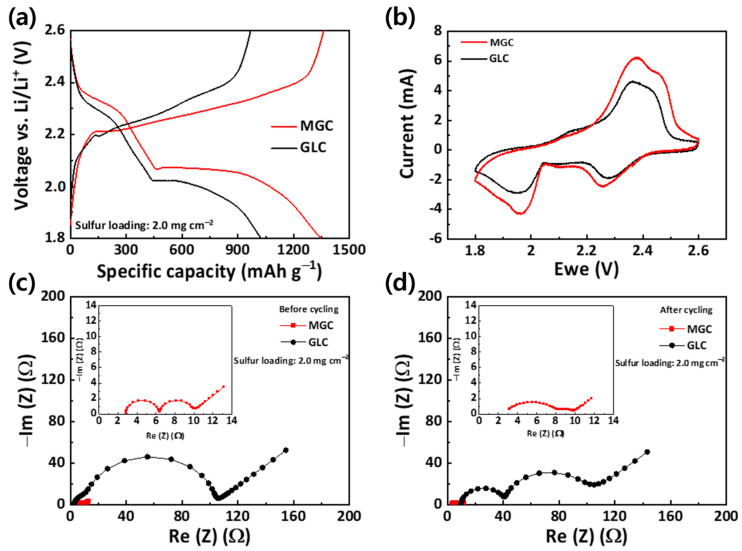
(**a**) Galvanostatic cycling with potential limitation (GCPL) profile at 1 mA cm^−2^ and 5th cycle, (**b**) cyclic voltammetry (CV) curves of GLC and MGC, electrochemical impedance spectra (EIS) of (**c**) GLC and MGC before cycling, (**d**) GLC and MGC after cycling.

Galvanostatic cycling with potential limitation (GCPL), rate capability, cycle voltammetry (CV), and electrochemical impedance spectroscopy (EIS) were conducted to identify the electrochemical performance of MGC as conductive sulfur supports. Figure 4a depicts that GLC generates only a specific capacity of 1023 mAh g^−1^, which is similar to previously reported CC as a carbon supports, whereas MGC generates 1383 mAh g^−1^ at a constant current density of 1 mA cm^−2^ [21]. This can be explained by the physical adsorption and capillary force induced from the meso-/micro-porosity of MGC, which make it adsorb LiPS efficiently [22]. The hierarchically porous structure of MGC enables the improvement in polysulfide trapping and further redox reactions of liquid to solid reaction, low polysulfide formation reaction; Li_2_S_x_ (x = 1 or 2), which is evidenced by the increased capacity of the low plateau region below 2.1 V vs. Li/Li^+^ in discharge profile. Figure 4b shows the CV curves of GLC and MGC in the range of 1.8–2.6 V vs. Li/Li^+^ at a scan rate of 0.1 mV s^−1^_,_ which indicates a similar result to GCPL. It seems that the first cathodic currents are almost similar due to the presence of CC current collector [21]. However, the second cathodic peaks have different currents: GLC; −2.88 mA and MGC; −4.35 mA. Moreover, MGC, with a higher surface area and pyridinic N content, enables serving enough space for the formation of Li_2_S and generation of additional capacity derived from the anchored LiPS.

The EIS results of GLC and MGC were fitted with the constant phase element (CPE) model and the Nyquist plots with two semicircles in the HF region (250 kHz–1 kHz), MF region (1 kHz–1 Hz), and one sloping line in the LF region (1 Hz–50 mHz) to investigate the electrochemical response of the carbon material (Figure 4c,d). Four types of resistance can be categorized from the model as follows: bulk cell resistance (R_b_), corresponding to the resistance of the electrolyte; interphase contact resistance (R_int_), reflecting the interphase electron conduction from current collector to reaction sites; charge-transfer resistance (R_ct_), representing the charge-transfer between the solid and liquid (conductive agent and electrolyte); and Warburg impedance (R_w_), which represents Li-ion diffusion (Table 3) [23]. R_int_ and R_ct_ of GLC (8.82 Ω and 90.43 Ω, respectively) are significantly higher than those of MGC (3.55 Ω and 3.27 Ω, respectively), indicating the improved electrical conductivity and wettability induced by the higher aromatic condensation and increased polarity with the introduction of nitrogen.

**Figure 5:**
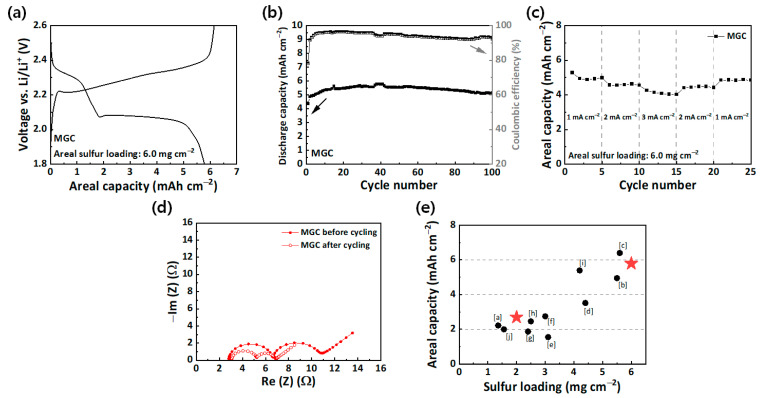
(**a**) GCPL profile of MGC under sulfur loading 6.0 mg cm^−2^ at 1 mA cm^−2^ and 39th cycle, (**b**) cycle stability, (**c**) rate capability, (**d**) EIS spectra, and (**e**) areal capacity vs. sulfur loading Ragone plot.

To achieve an areal capacity higher than those of the commercially available LIBs (4 mAh cm^−2^), electrochemical analyses were conducted under a high areal sulfur loading of 6.0 mg cm^−2^. The MGC is little passivated by the charge/discharge product (S_8_ and Li_2_S) under the high sulfur loading condition, showing minimal change in polarization between charge and discharge profile (Figure 5a). It exhibits a gradual activation at initial cycles and a maximum areal capacity of 5.79 mAh cm^−2^ at the 39th cycle. The cycle stability is maintained over 100 cycles with a capacity decay rate per cycle of 0.18% (Figure 5b). N-containing porous carbon, which has an affinity to LiPS, suppresses the shuttle effect, and thus suffers no severe degradation of sulfur utilization. The rate capability investigated with galvanostatic charge and discharge with 1, 2, and 3 mA cm^−2^ is illustrated in Figure 5c. At 2 and 3 mA cm^−2^, the discharge capacity was generated at 91.2% and 81.1%, respectively, of that of 1 mA cm^−2^. The three-point-contact among the electrolyte, electrons, and active materials is facilitated, thereby preventing severe discharge capacity loss even at the high current densities. It retains 97.2% of the initial discharge capacity at 1 mA cm^−2^ after several current density alterations. Before and after cycling, R_int_ and R_ct_ of MGC decrease from 3.77 Ω and 3.78 Ω to 2.18 Ω and 1.53 Ω, respectively (Figure 5d, Table 4).

The Ragone plot to compare this material with variable carbon-based sulfur host materials is illustrated in Figure 5e. The electrochemical performance obtained in this study (areal sulfur loading of 6.0 mg cm^−2^ and areal capacity of 5.79 mAh cm^−2^) is remarkable. Figure 5e and Table 5 summarize the electrochemical performance, including sulfur loading, maximum areal capacity, sulfur utilization, capacity decay rate, and cycle stability, of the recently reported carbon-based sulfur cathode in LSBs, are provided. In this work, MGC-based LSB cells achieved a sulfur utilization efficiency of 93.60% and 57.71% at an areal sulfur loading of 2.0 mg cm^−2^ and 6.0 mg cm^−2^, respectively. It can be seen that these values are superior or comparable to those of the current state-of-the-art LSBs. We note that the enhanced LiPS affinity resulting from rationally hierarchical pore structure and enough pyridinic N species leads to excellent sulfur utilization and cycle stability with a minor capacity decay rate per cycle.

## 4. Conclusions

In conclusion, this study proposes multi-functional carbon-based sulfur host material supports to suppress the polysulfide dissolution and generate high areal capacity in LSBs. To achieve these, (1) hierarchically porous structure, (2) pyridinic N-functional groups, and (3) more aromatically condensed structure possessing carbon is prepared. This was obtained through the simple mixing of glucose as a carbon source and MCA as a reactive template and then by carbonization of the mixture. Although MGC had a high nitrogen content of up to 7.59%, the sp^2^-hybridized 2D structure was well-linked without severe detriment, resulting in low charge transfer resistance (3.27 Ω). With high loading sulfur cathode in LSBs, MGC exhibited a good ability toward the physi-/chemisorption of soluble LiPS, and thus the improved high areal capacity of 5.79 mAh cm^−2^ was obtained at a constant areal current density of 1 mA cm^−2^ with a capacity decay rate of 0.18% per cycle over 100 cycles. This study presents an efficient way to prepare carbon supports for high-sulfur-loaded LSBs while effectively suppressing the shuttle effect.

## Figures and Tables

**Table 1:** Crystal structure of GLC and MGC; interlayer space, Lc, La, stacking number, R factor, and I_G_/I_D_.

Materials	*d_002_* (nm)	(002) Peak Position (2 theta)	L_c_ (nm)	L_a_ (nm)	Stacking Number	R factor	I_G_/I_D_
GLC	0.42	21.15	1.22	2.45	3.89	1.85	1.05
MGC	0.40	22.41	0.92	2.72	3.33	1.91	1.00

**Table 2:** The elemental weight percentage of elemental analysis.

Materials	Carbon (wt.%)	Nitrogen (wt.%)	Hydrogen (wt.%)	Oxygen (wt.%)
GLC	89.16	0.28	2.41	2.42
MGC	71.44	11.69	2.97	8.01

**Table 3:** EIS fitting results using a CPE model.

Cell	R_b_ (Ω)	R_int_ (Ω)	R_ct_ (Ω)	R_w_ (Ω^−^^0.5^)
GLC-before	2.71	8.82	90.43	29.41
MGC-before	2.79	3.55	3.27	1.97

**Table 4:** EIS fitting results of MGC using a CPE model under sulfur loading 6.0 mg cm^−2^.

Cell	R_b_ (Ω)	R_int_ (Ω)	R_ct_ (Ω)	R_w_ (Ω^−^^0.5^)
MGC before cycling	2.80	3.77	3.78	1.78
MGC after cycling	3.04	2.18	1.53	0.99

**Table 5:** Electrochemical performances of present studies with tuning cathode.

Label	Sulfur Loading (mg cm^−2^)	Maximum Areal Capacity (mAh cm^−2^)	Sulfur Utilization(%)	Capacity Decay Rate (% per cycle)	Cycle Number	Reference
[a]	1.36	2.22	97.63	0.27	100	[24]
[b]	5.5	4.95	53.83	0.042	1200	[25]
[c]	5.6	6.4	68.35	0.472	100	[26]
[d]	4.4	3.82	51.92	0.55	100	[27]
[e]	3.1	2.07	39.94	0.05	500	[28]
[f]	3.2	2.93	54.76	0.23	100	[29]
[g]	2.4	2.45	61.05	0.09	100	[30]
[h]	2.5	2.46	58.85	0.13	100	[31]
[i]	4.2	5.4	76.90	0.051	100	[32]
[j]	2.0	1.56	46.65	0.36	100	[33]
This work	2.0	3.13	93.60	0.21	100	**-**
This work	6.0	5.79	57.72	0.18	100	**-**

## References

[1] Choi J.W., Aurbach D. (2016). Promise and reality of post-lithium-ion batteries with high energy densities. Nat. Rev. Mater..

[2] Marom R., Amalraj S.F., Leifer N., Jacob D., Aurbach D. (2011). A review of advanced and practical lithium battery materials. J. Mater. Chem..

[3] Bruce P.G., Freunberger S.A., Hardwick L.J., Tarascon J.-M. (2012). Li–O2 and Li–S batteries with high energy storage. Nat. Mater..

[4] Chen X., Hou T., Persson K.A., Zhang Q. (2019). Combining theory and experiment in lithium–sulfur batteries: Current progress and future perspectives. Mater. Today.

[5] Rana M., Ahad S.A., Li M., Luo B., Wang L., Gentle I., Knibbe R. (2019). Review on areal capacities and long-term cycling performances of lithium sulfur battery at high sulfur loading. Energy Storage Mater..

[6] Kim J.H., Lee Y.H., Cho S.J., Gwon J.G., Cho H.J., Jang M., Lee S.Y. (2019). Nanomat Li-S batteries based on all-fibrous cathode/separator assemblies and reinforced Li metal anodes: Towards ultrahigh energy density and flexibility. Energy Environ. Sci..

[7] Ogoke O., Wu G., Wang X., Casimir A., Ma L., Wu T., Lu J. (2017). Effective strategies for stabilizing sulfur for advanced lithium–sulfur batteries. J. Mater. Chem. A.

[8] Chung S.-H., Han P., Singhal R., Kalra V., Manthiram A. (2015). Electrochemically Stable Rechargeable Lithium–Sulfur Batteries with a Microporous Carbon Nanofiber Filter for Polysulfide. Adv. Energy Mater..

[9] Manthiram A., Fu Y., Chung S.-H., Zu C., Su Y.-S. (2014). Rechargeable Lithium–Sulfur Batteries. Chem. Rev..

[10] Zhang B., Qin X., Li G.R., Gao X.P. (2010). Enhancement of long stability of sulfur cathode by encapsulating sulfur into micropores of carbon spheres. Energy Environ. Sci..

[11] Ji X.L., Evers S., Black R., Nazar L.F. (2011). Stabilizing lithium-sulphur cathodes using polysulphide reservoirs. Nat. Commun..

[12] Eftekhari A., Kim D.-W. (2017). Cathode materials for lithium–sulfur batteries: A practical perspective. J. Mater. Chem. A.

[13] Dong C., Gao W., Jin B., Jiang Q. (2018). Advances in Cathode Materials for High-Performance Lithium-Sulfur Batteries. iScience.

[14] Wang M., Xia X., Zhong Y., Wu J., Xu R., Yao Z., Wang D., Tang W., Wang X., Tu J. (2019). Porous Carbon Hosts for Lithium–Sulfur Batteries. Chem. Eur. J..

[15] Kang H.-J., Huh Y.S., Im W.B., Jun Y.-S. (2019). Molecular Cooperative Assembly-Mediated Synthesis of Ultra-High-Performance Hard Carbon Anodes for Dual-Carbon Sodium Hybrid Capacitors. ACS Nano.

[16] Jun Y.-S., Lee E.Z., Wang X., Hong W.H., Stucky G.D., Thomas A. (2013). From Melamine-Cyanuric Acid Supramolecular Aggregates to Carbon Nitride Hollow Spheres. Adv. Funct. Mater..

[17] Olejniczak A., Tomala R., Cichy B., Głuchowski P., Jakimów M., Zięba A., Kępiński L., Ignatenko O., Stręk W. (2019). Laser-driven proliferation of sp2-sp3 changes during anti-Stokes white light emission of μ-diamonds. Carbon.

[18] Yin L.-C., Liang J., Zhou G.-M., Li F., Saito R., Cheng H.-M. (2016). Understanding the interactions between lithium polysulfides and N-doped graphene using density functional theory calculations. Nano Energy.

[19] Wang H., Maiyalagan T., Wang X. (2012). Review on Recent Progress in Nitrogen-Doped Graphene: Synthesis, Characterization, and Its Potential Applications. ACS Catal..

[20] Jena H.S., Krishnaraj C., Parwaiz S., Lecoeuvre F., Schmidt J., Pradhan D., Van Der Voort P. (2020). Illustrating the Role of Quaternary-N of BINOL Covalent Triazine-Based Frameworks in Oxygen Reduction and Hydrogen Evolution Reactions. ACS Appl. Mater. Interfaces.

[21] Song J.-Y., Lee H.-H., Hong W., Huh Y., Lee Y., Kim H., Jun Y.-S. (2018). A Polysulfide-Infiltrated Carbon Cloth Cathode for High-Performance Flexible Lithium–Sulfur Batteries. J. Nanomater..

[22] Fang Z., Luo Y., Wu H., Yan L., Zhao F., Li Q., Fan S., Wang J. (2020). Mesoporous carbon nanotube aerogel-sulfur cathodes: A strategy to achieve ultrahigh areal capacity for lithium-sulfur batteries via capillary action. Carbon.

[23] Deng Z., Zhang Z., Lai Y., Liu J., Li J., Liu Y. (2013). Electrochemical Impedance Spectroscopy Study of a Lithium/Sulfur Battery: Modeling and Analysis of Capacity Fading. J. Electrochem. Soc..

[24] Jin F., Xiao S., Lu L., Wang Y. (2016). Efficient Activation of High-Loading Sulfur by Small CNTs Confined Inside a Large CNT for High-Capacity and High-Rate Lithium-Sulfur Batteries. Nano Lett..

[25] Liang X., Rangom Y., Kwok C.Y., Pang Q., Nazar L.F. (2017). Interwoven MXene Nanosheet/Carbon-Nanotube Composites as Li-S Cathode Hosts. Adv. Mater..

[26] Zhang S., Zhong N., Zhou X., Zhang M., Huang X., Yang X., Meng R., Liang X. (2020). Comprehensive Design of the High-Sulfur-Loading Li–S Battery Based on MXene Nanosheets. Nano-Micro Lett..

[27] Li Q., Guo J., Zhao J., Wang C., Yan F. (2019). Porous nitrogen-doped carbon nanofibers assembled with nickel nanoparticles for lithium–sulfur batteries. Nanoscale.

[28] Liu H., Thomas T., Li R., Shen H., Wang J., Yang M. (2020). Multifunctional hosts of Zinc sulfide coated carbon nanotubes for lithium sulfur batteries. SN Appl. Sci..

[29] Xie K.Y., Zhang K., Han Y.Z., Yuan K., Song Q., Wang J.G., Shen C., Liu X.R., Wei B.Q. (2016). A Novel TiO_2_-Wrapped Activated Carbon Fiber/Sulfur Hybrid Cathode for High Performance Lithium Sulfur Batteries. Electrochim. Acta.

[30] Xu H., Qie L., Manthiram A. (2016). An integrally-designed, flexible polysulfide host for high-performance lithium-sulfur batteries with stabilized lithium-metal anode. Nano Energy.

[31] Ye X., Ma J., Hu Y.-S., Wei H., Ye F. (2016). MWCNT porous microspheres with an efficient 3D conductive network for high performance lithium–sulfur batteries. J. Mater. Chem. A.

[32] Peng H.J., Xu W.T., Zhu L., Wang D.W., Huang J.Q., Cheng X.B., Yuan Z., Wei F., Zhang Q. (2016). 3D Carbonaceous Current Collectors: The Origin of Enhanced Cycling Stability for High-Sulfur-Loading Lithium-Sulfur Batteries. Adv. Funct. Mater..

[33] Kang H.-J., Bari G.A.K.M.R., Lee T.-G., Khan T.T., Park J.-W., Hwang H.J., Cho S.Y., Jun Y.-S. (2020). Microporous Carbon Nanoparticles for Lithium–Sulfur Batteries. J. Nanomat..

